# Detecting recurrent prostate Cancer using multiparametric MRI, influence of PSA and Gleason grade

**DOI:** 10.1186/s40644-020-00373-4

**Published:** 2021-01-06

**Authors:** Aradhana M. Venkatesan, Eniola Mudairu-Dawodu, Cihan Duran, R. Jason Stafford, Yuanqing Yan, Wei Wei, Vikas Kundra

**Affiliations:** 1 Department of Diagnostic Radiology, Division of Diagnostic Radiology, MD Anderson Cancer Center, Houston, TX USA; 2 West Houston Radiology Associates, 21216 North West Freeway, Suite 2200, Cypress, TX USA; 3 Department of Diagnostic and Interventional Imaging, UT Houston, 6411 Fannin Street, Suite J2.222, Houston, TX USA; 4 Department of Imaging Physics, Division of Diagnostic Imaging, The University of Texas MD Anderson Cancer Center, Houston, TX USA; 5 Department of Biostatistics, MD Anderson Cancer Center, Houston, TX USA; 6 Department of Cancer Systems Imaging, Division of Diagnostic Radiology, MD Anderson Cancer Center, Houston, TX USA

**Keywords:** Prostate cancer, Recurrence, Multiparametric MRI, Radical prostatectomy

## Abstract

**Background:**

The utility of multiparametric MRI (mpMRI) in detecting suspected local recurrence post radical prostatectomy (RP) may be associated with PSA and Gleason grade. The purpose of the study was to evaluate the likelihood of detecting locally recurrent prostate cancer utilizing mpMRI in patients with suspected recurrence following radical prostatectomy (RP) parsed by PSA and Gleason grade.

**Methods:**

One hundred ninety five patients with suspected local recurrence were imaged on a 1.5 T MRI with torso array and endorectal coil in this retrospective study. mpMRI interpretations were stratified by PSA and lower (Gleason < 7) vs. higher grade tumors (Gleason 8–10). Recursive partitioning was used to determine whether mpMRI interpretations could be classified as positive or negative.

**Results:**

The majority of mpMRI interpretations in patients with lower Gleason grade tumors and PSA < 0.5 ng/mL were negative (68/78, 87.2%, *p* = 0.004). The majority of mpMRI interpretations in patients with higher Gleason grade tumors and PSA > 1.5 ng/mL were positive (8/9, 88.9%, *p* = 0.003). Findings were corroborated by recursive partitioning, which identified a PSA = 0.5 ng/ml in patients with lower grade tumors and a PSA = 1.5 ng/mL in patients with higher grade tumors as differentiating negative and positive mpMRIs.

**Conclusion:**

In the setting of suspected recurrence after RP, mpMRI results are associated with PSA and Gleason grade, both of which can help guide when mpMRI may find utility. mpMRI is likely to be low diagnostic yield and negative for recurrence (87%) in the setting of lower Gleason grade tumors and PSA < 0.5 ng/mL. mpMRI is likely to be of low diagnostic value and positive for recurrence (89%) in the setting of PSA > 1.5 ng/mL and higher grade tumors; in this case, mpMRI findings may be more useful for directing biopsy and local therapy. Between these extremes, PSA > 0.5 ng/mL and lower grade tumors or PSA < 1.5 ng/mL and higher grade tumors, mpMRI results are less predictable, suggesting greater diagnostic value for detecting recurrence post prostatectomy.

## Background

Prostate cancer is the most common cancer in men in the United States and is the second leading cause of death from cancer [1]. Cancer localized to the prostate at diagnosis is treated primarily with radical prostatectomy (RP), external beam radiation, or brachytherapy with or without hormone ablation therapy. In the United States, approximately 40% of the patients undergo RP with curative intent. After RP, prostate-specific antigen (PSA) drops to zero. Unfortunately, after RP, 20–40% of patients develop biochemical recurrence within 10 years with a median time of 20–38 months. Diagnosis and management of local recurrence of prostate cancer following RP remains an ongoing clinical challenge [2]. Currently, diagnosis is based on the onset of biochemical recurrence (BCR), commonly defined as two post-treatment PSA values > 0.2 ng/mL [3]. Evidence level is C, conditional, and clinicians may suspect BCR at lower PSA [2]. However, BCR is not synonymous with local recurrence in the prostatic bed, and can be due to distant metastases, local disease, or both. Distant bone metastases can be evaluated by bone scan and whole body MR including diffusion weighted imaging [4], and, is now increasingly assessed using PET/CT [2, 5]. Moreover, a persistently elevated serum PSA level may be due to residual healthy glandular tissue [6].

Historically, detection of local recurrence post RP has been a challenge. Given typically low tumor volume initially at relapse, BCR is rarely accompanied by a detectable mass at digital rectal examination and is difficult to detect by transrectal ultrasound guided biopsy [6]. Therapy is typically initiated at a PSA of 0.4 ng/mL or higher [3, 7–9]. Early detection is important for subsequent management because local recurrence may be approached using local interventions such as surgery, radiation therapy and cryoablation, whereas, distant metastases require systemic therapy.

Multiparametric MRI (mpMRI) is one of the most useful imaging modalities for detection and localization of local prostate cancer recurrence after RP [6, 10]. It offers high tissue contrast and spatial resolution of the pelvis compared to other imaging modalities and supplies both anatomic and functional information regarding the prostatectomy bed as well as pelvic lymph nodes and bones using sequences such as T2-weighted morphologic assessment, dynamic contrast-enhanced (DCE) imaging, and diffusion-weighted imaging (DWI) [6, 10]. However, given the wide range in tumor aggressiveness and variability in PSA kinetics following RP, it remains unclear which patients with an elevated PSA following RP may most benefit from mpMRI assessment. The objective of this study was to evaluate the likelihood of detecting locally recurrent prostate cancer using mpMRI in patients with suspected local recurrence following RP, parsed by PSA and tumor Gleason grade.

## Methods

### Patient population

**Fig. 1:**
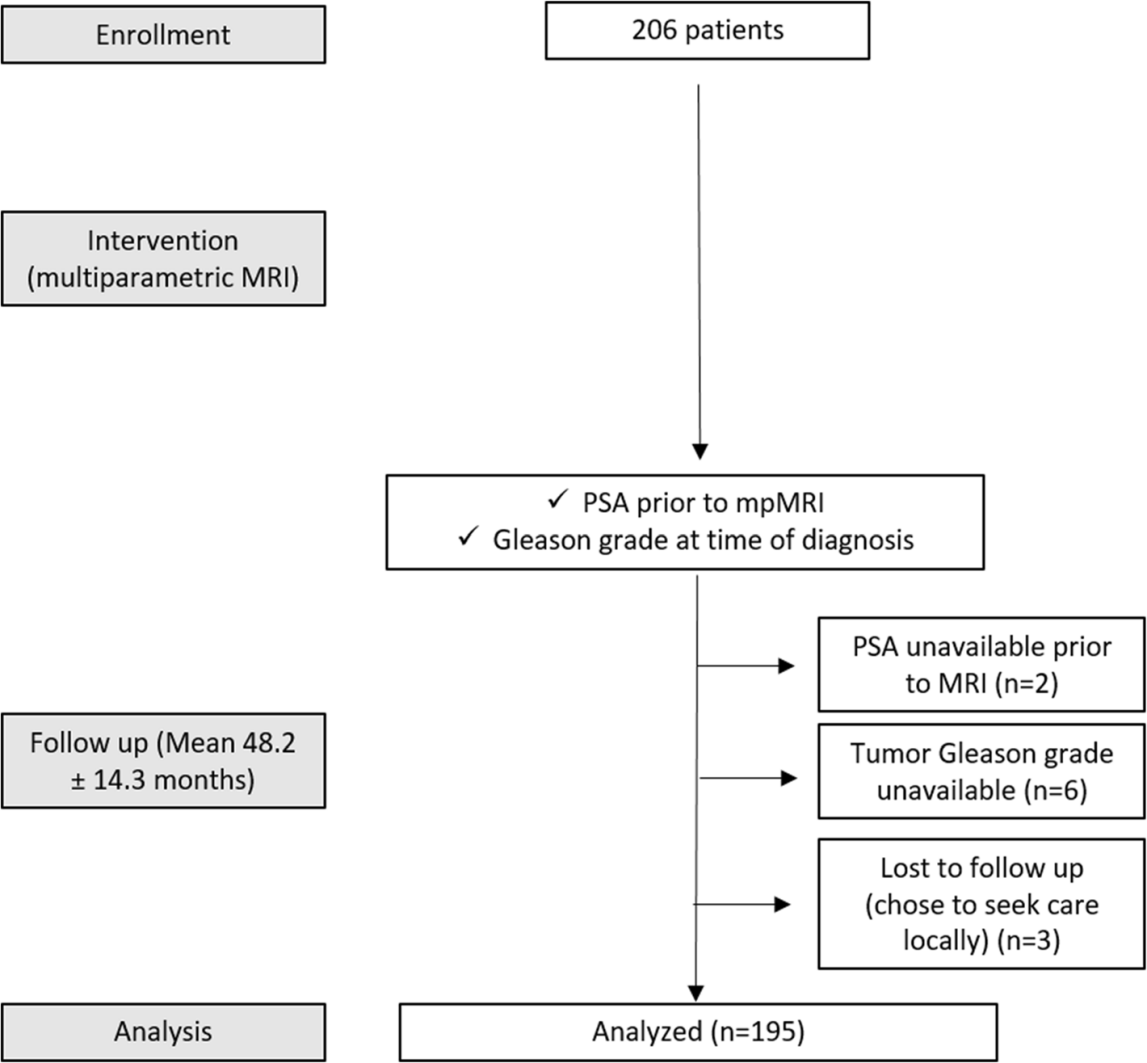
CONSORT diagram

This HIPAA compliant, single-institution retrospective study of prospectively acquired data received approval by the local institutional review board. No written informed consent was necessary. The study cohort comprised 195 men (mean age = 64.4 years; range 42–78 years) who underwent mpMRI in 2012–2014 for suspicion of local recurrence (i.e. PSA >  0) after previous RP, and had both Gleason score and PSA available (Fig. 1). Patients were enrolled consecutively. PSA values were available within a month of the mpMRI. Patients who elected to pursue follow-up care locally post mpMRI, i.e. for whom follow-up information was unavailable, were excluded (*n* = 3). Patients without available contemporaneous PSA (*n* = 2) or Gleason score (*n* = 6) were excluded. To avoid bias, patients were not parsed by clinical risk groups. Clinical records were reviewed for subject demographic data, including age; PSA; and Gleason grade. Patients were pathologically staged according to the 2010 American Joint Committee on Cancer staging system.

### Imaging methods

1.5 T MR imaging was performed using an endorectal coil. mpMRI images included axial T1W FSE of the pelvis (TR = 750–900 ms; TE = minimum full; echo train length (ETL) = 3; receiver bandwidth (rBW) = + 31.25 kHz; matrix = 320 × 224; field of view (FOV) = 24 cm; slice thickness = 5 mm skip 1 mm; number of excitations (NEX) = 2) as well as whole pelvis DWI using a torso array (b-values = 100,700 s/mm^2^; TR = 8000 ms; TE = minimum; matrix = 100 × 160; FOV = 38 cm; slice thickness = 5 mm skip 1 mm; NEX = 2/6). A pelvic phased array and endorectal coil were used for high resolution imaging of the prostatectomy bed. High resolution orthogonal plane (axial, sagittal, coronal) T2W FSE images were obtained (TR = 6000–10,000 ms; TE = 200 ms; ETL = 21; rBW = + 31.25 kHz; matrix = 320 × 224; FOV = 14–16 cm; slice thickness = 3 mm skip 0 mm; NEX = 4) as well as axial T1W FSE (TR = 750–900 ms; TE = minimum full; ETL = 3; rBW = + 20.83 kHz; matrix = 320 × 224; FOV = 14 cmx8cm; slice thickness = 3 mm skip 0 mm) and axial DWI (b-values = 50, 700 s/mm^2^; TR = 8000 ms; TE = minimum; matrix = 100 × 160; FOV = 14 cm × 8 cm; slice thickness = 3 mm skip 0 mm; NEX = 2/4/16). ADC reconstructions were performed. A 3D fast radiofrequency-spoiled gradient-recalled echo (FSPGR) sequence (TR = minimum; TE = 1.2 ms; rBW = + 41.67 kHz; matrix = 192 × 128; FOV = 22 cm; slice thickness = 3 mm skip − 1.5 mm) was acquired during contrast uptake at a rate of ≤10 s per volume over 5 min (22–26 time points) after injection of 0.1 mmol/kg gadopentetate dimeglumine (Magnevist, Schering AG, Berlin, Germany), at a rate of 3 mL/second.

### Clinical data analysis

Study subjects’ radiology reports were reviewed. Interpretations were made in a clinical setting with all data in the chart available including history and laboratory findings by fellowship trained radiologists focused on abdomen/pelvis imaging in a tertiary care hospital focused on cancer. The radiology reports were classified based upon the impression into categories of definite recurrence, possible recurrence, no definite recurrence and no recurrence. Impressions classified as definite and possible recurrences were scored as positive studies; those read as no definite recurrence or no recurrence were scored as negative studies. These mpMRI interpretations were used for statistical analyses. Mean follow-up after MR imaging was 48.2 ± 14.3 months and included biopsy, follow-up imaging, or clinical disposition. Among positive MRI reads, recurrence was confirmed with follow-up biopsy in 19 subjects, 15 underwent salvage radiation therapy in the absence of histologic confirmation with subsequent decrease in PSA, 13 were positive by clinical disposition or follow-up imaging and 4 were negative by clinical disposition and among the latter, PSA was 0.05, 0.1, 0.3, and 3.7 ng/ml with low, high, high, and low grade tumors respectively.

### Statistical analyses

The difference in PSA between positive and negative mpMRI interpretations for lower (Gleason < 7) and higher grade tumors (Gleason 8–10) was evaluated with the Wilcoxon rank sum test. Recursive partitioning and regression trees (RPART) were utilized to determine the optimal PSA cutoff for patients with higher and lower grade tumors [11]. Wilcoxon rank sum test and Fisher’s exact test assessed association between different factors. Statistical analyses were performed using SAS version 9.3 (SAS Institute, Cary, NC) and R version 3.1.1 (R Foundation, Vienna, Austria). All tests were two-sided and a *p-*value < 0.05 was considered statistically significant.

## Results

**Fig. 2:**
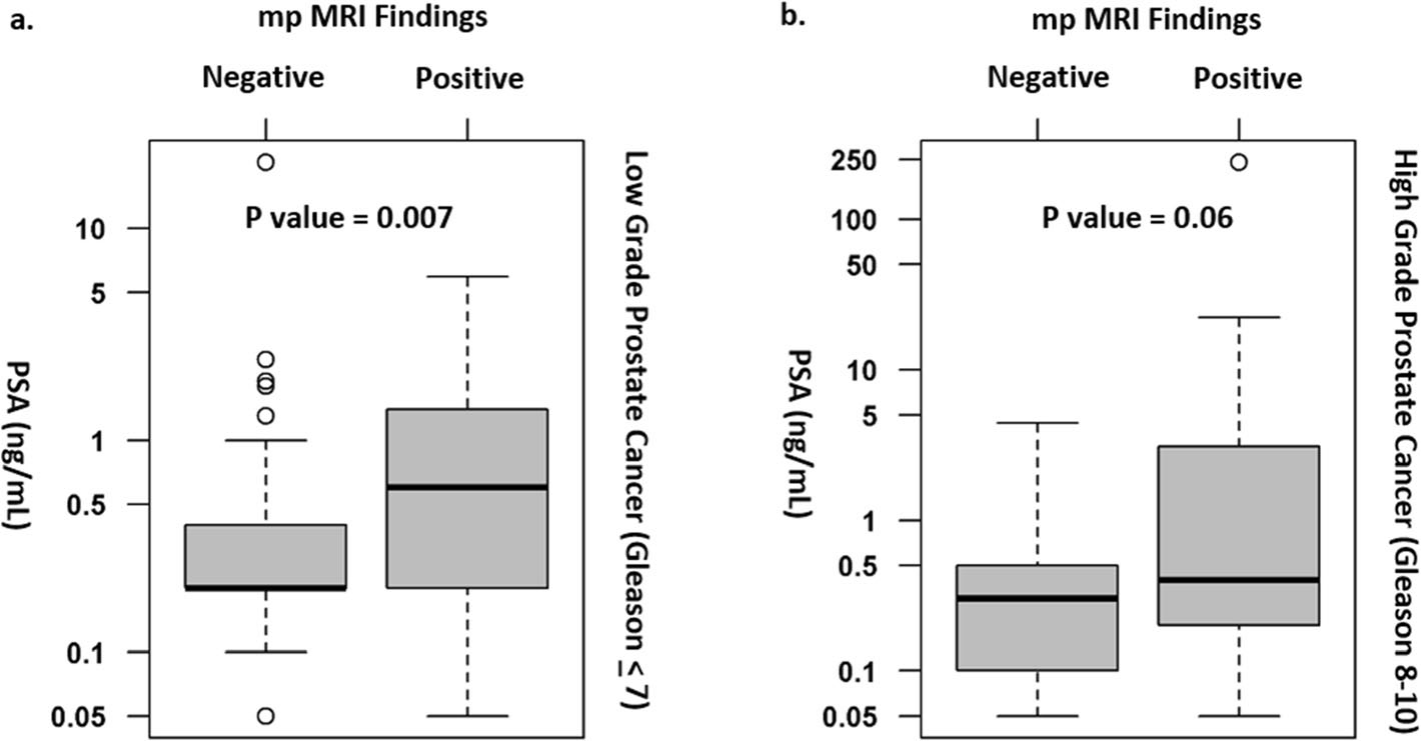
Boxplot depiction of mpMRI results as a function of PSA level for (**a**) lower grade tumors (Gleason < 7) and (**b**) higher grade tumors (Gleason 8–10), (Bolded vertical line delineates median value, open circles denote outliers). Note that the x-axis is non-linear

Subject demographics are presented in Table 1. The PSA level for patients with a positive mpMRI was significantly higher than with a negative mpMRI for lower grade tumors (*p* = 0.007) (Table 2 and Fig. 2) and approached significance for higher grade tumors (*p* = 0.06) (Table 2 and Fig. 2b).

**Table 1:**
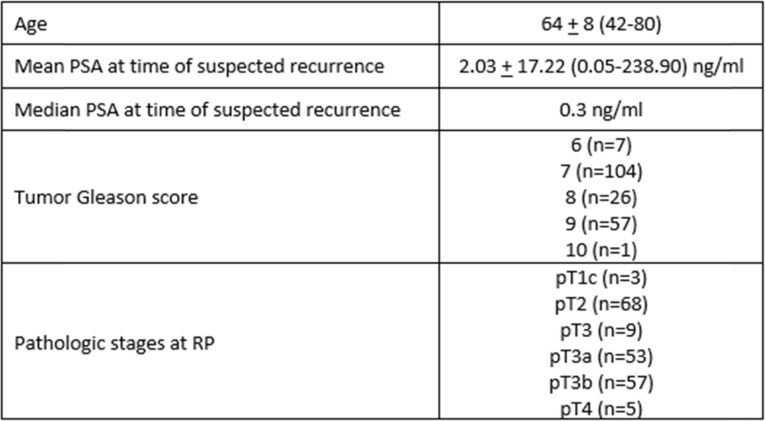
Subject Demographics (*n* = 195).

*Subjects not sub-classified by pathologist as either pT3a or pT3b

**Table 2:** mpMRI interpretation, tumor Gleason grade and PSA level for patients with suspected recurrence after RP

		N	Mean	SD	Minimum	Median	Maximum	***P***-value
			PSA (ng/ml)		PSA (ng/ml)	PSA (ng/ml)	PSA (ng/ml)	
**Gleason Score**	**mpMRI result**							
**Lower (< 7)**	**Negative**	**86**	**0.6**	**2.21**	**0.05**	**0.2**	**20.5**	**0.007**
	**Positive**	**25**	**0.98**	**1.31**	**0.05**	**0.6**	**5.9**	
**Higher (8–10)**	**Negative**	**58**	**0.4**	**0.6**	**0.05**	**0.3**	**4.4**	**0.06**
	**Positive**	**26**	**11.43**	**46.64**	**0.05**	**0.4**	**238.9**

Based upon initial evaluation of the data, a PSA cutoff of 0.5 ng/mL for lower grade tumors was estimated as the optimal cutoff to partition mpMRI results into either negative (PSA <  0.5 ng/mL) or positive (PSA > 0.5 ng/mL). This subjective assessment was tested and confirmed for lower grade tumors, with the majority of those patients with a PSA <  0.5 ng/mL having a negative mpMRI (68/78, 87.2%, *p* = 0.0004) (Table 3). This was not seen for higher grade tumors (*p* = 0.32). A PSA value of 1.5 ng/mL was estimated as the optimal cutoff for higher grade tumors. This subjective assessment was also tested and confirmed, with the majority of patients with higher grade tumors and a PSA > 1.5 ng/mL having a positive mpMRI (8/9, 88.9%, *p* = 0.0003). Patients with lower grade tumors and a PSA > 0.5 ng/mL and patients with higher grade tumors and PSA < 1.5 ng/ml were less predictable (Table 3).

**Table 3:** mpMRI interpretation, tumor Gleason grade and PSA level for patients with suspected recurrence after RP with estimated optimal PSA cutoffs

	PSA at Recurrence	mpMRI interpretation				***P*** = value
		Negative		Positive		
**Gleason Score**	**ng/ml**	**N**	**%**	**N**	**%**	
**Lower (< 7)**	**< 0.5**	**68**	**87.18**	**10**	**12.82**	**0.0004**
	**> 0.5**	**18**	**54.55**	**15**	**45.45**	
**Higher (8–10)**	**< 0.5**	**41**	**73.21**	**15**	**26.79**	**0.32**
	**> 0.5**	**17**	**60.71**	**11**	**39.29**	
	**< 1.5**	**57**	**76**	**18**	**24**	**0.0003**
	**> 1.5**	**1**	**11.11**	**8**	**88.89**	

**Fig. 3:**
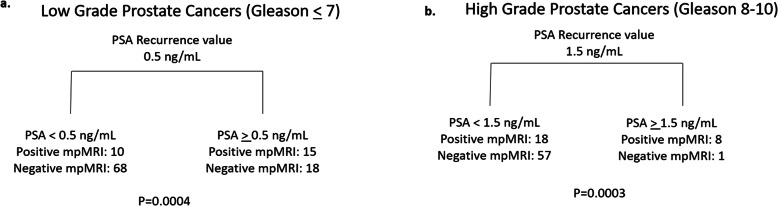
Optimal PSA cutoffs for (**a**) lower and (**b**) higher grade Gleason tumors as assessed by Recursive Partitioning and Regression Trees

**Fig. 4:**
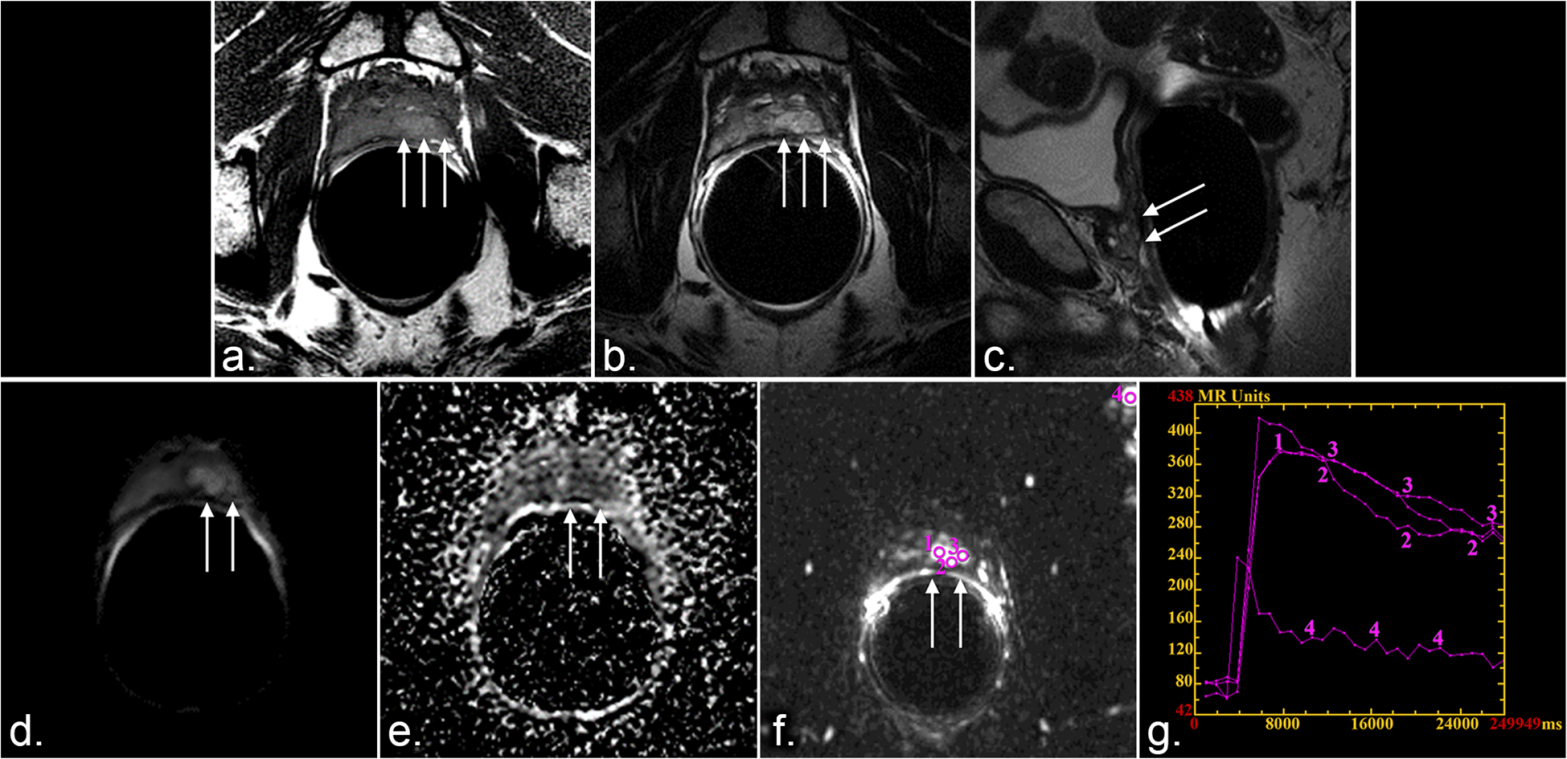
Positive mpMRI in patient with high grade Gleason tumor and elevated PSA (> 1.5 ng/mL) after RP. 69 year old male treated with radical prostatectomy for T3a, Gleason 8 adenocarcinoma presented with BCR after RP (PSA 5.2 ng/mL). Multiparametric MRI including axial T1 (**a**), axial T2 (**b**), sagittal T2 (**c**) axial diffusion weighted images (**d**), axial apparent diffusion coefficient (ADC) images (**e**), dynamic contrast enhanced (DCE) MRI (**f**) images and contrast enhancement time curves (**g**) reveal confluent T2 hyperintense soft tissue at the level of the vesicourethral anastomosis, consistent with residual prostate tissue. Additionally, an eccentric T1 heterogeneous, T2 hyperintense mass involving the left lateral aspect of the vesicourethral anastomosis is observed (arrows in **a, b**), extending into the peri-anastomotic soft tissues (arrows in **c**). The mass is associated with restricted diffusion (arrows in **d** and **e**) and focal enhancement (arrows in **f**). Contrast enhancement time curves (**g**) demonstrate a pattern of rapid contrast wash in and washout associated with malignancy (curves 1, 2, and 3 = tumor, curve 4 = arterial). Recurrent tumor was confirmed by transurethral biopsy

These data were further validated via Recursive Partitioning and Regression Trees (RPART), a non-parametric regression and classification statistical method. Use of RPART confirmed the optimal PSA cutoff value to be < 0.5 ng/mL to distinguish likely negative mpMRI studies in patients with lower Gleason grade tumors (Fig. 3a). An optimal PSA cutoff value of > 1.5 ng/mL distinguished likely positive mpMRI studies in patients with high Gleason grade tumors, with the majority of mpMRI studies in these patients being positive (Fig. 3b, Fig. 4).

Clinical investigations are typically initiated at a PSA of 0.2 ng/mL [3], with therapy typically initiated at a PSA of 0.4 ng/mL or higher [3, 7, 8], however, we did not observe a statistically significant difference at these values except for at a lower Gleason grade (< 7) and PSA value of 0.4 ng/mL (*p* = .02) (Table 4). As expected from above, most (~ 85%) patients with a lower Gleason grade and PSA < 0.2 or < 0.4 ng/mL had a negative mpMRI interpretation.

**Table 4:** mpMRI interpretation, tumor Gleason grade and PSA level for patients with suspected recurrence after RP using conventional 0.2 and 0.4 ng/mL PSA cutoffs

		mpMRI interpretation				***P*** = value
		Negative		Positive		
**Gleason Score**	**PSA**	**N**	**%**	**N**	**%**	
**Lower (< 7)**	**< 0.2**	**18**	**85.71**	**3**	**14.29**	**0.40**
	**> 0.2**	**68**	**75.56**	**22**	**24.44**	
	**< 0.4**	**59**	**85.51**	**10**	**14.49**	**0.02**
	**> 0.4**	**27**	**64.29**	**15**	**35.71**	
**Higher (8–10)**	**< 0.2**	**17**	**80.95**	**4**	**19.05**	**0.28**
	**> 0.2**	**41**	**65.08**	**22**	**34.92**	
	**< 0.4**	**37**	**75.51**	**12**	**24.49**	**0.16**
	**> 0.4**	**21**	**60**	**14**	**40**	

## Discussion

mpMRI results in the setting of suspected local recurrence after RP are associated with PSA and Gleason grade. BCR following RP is common, developing in approximately 50% of high risk patients and 10% of low risk patients within 15 years of surgery, often without clinical or radiological evidence of disease [10, 12]. Locally advanced disease is associated with increased risk of recurrence in the setting of extraprostatic extension, perineural invasion, seminal vesicle invasion, larger tumor size, lymphovascular invasion, and positive surgical margins [13]. Absence of a prostatic pseudocapsule at the apex as well as the desire to preserve the urethral sphincter and neurovascular bundles are thought to contribute to local recurrence [14]. In addition, recurrence in the pelvis may also occur in lymph nodes and bones. Freedland et al. found that BCR can precede clinical relapse by a median of 5 years and that the time to BCR after surgery has predictive value for cancer specific survival [15]. The rate of PSA elevation can suggest local or distant disease; for example, low velocity and long doubling time (> 6 months, range ~ 3–12 months in different studies) suggests local recurrence, whereas, the opposite suggests distant metastases [16, 17]. For the latter, nuclear medicine imaging, such as with radiolabeled choline, amino acid transport substrates, and prostate specific membrane antigen ligands, is now often used but has low sensitivity with PSA < 2 ng/ml and in particular with PSA <  1 ng/ml [18]; and, mpMRI has been suggested to have superior performance for local recurrence [5, 19]. Early detection is important because local recurrence post RP may be approached using local interventions, such as surgery, radiation therapy and cryoablation + hormonal therapy. Although nomograms have been developed, which patients will benefit from early treatment such as salvage radiotherapy needs further definition [20].

Several prior studies have evaluated the added value of mpMRI for detecting pelvic prostate cancer recurrence [10, 21–26]; however, to our knowledge, few have parsed these data both in terms of PSA level and tumor Gleason grade. Using 3 T mpMRI without an endorectal coil, Couñago et al. found that among 57 patients with BCR post RP, 14 (24.56%) had local recurrence reported via mpMRI, with the probability of local recurrence being significantly higher in patients with PSA levels above 0.5 ng/mL [27]. Kitajima et al. studied 80 men with suspected local recurrence post RP using 3 T mpMRI with an endorectal coil and found a higher likelihood for local recurrence detection in patients with PSA > 1.0 ng/mL versus < 1.0 ng/mL [28]; however, no stratification between patients with lower and higher Gleason grade tumors was performed. In contrast, Liauw et al., evaluating patients scanned at either 1.5 T (*n* = 59) or 3.0 T (*n* = 29) with an endorectal coil [29], found local recurrence in 24% (*n* = 21) and noted that PSA was an associated risk factor; in addition, they noted recurrence in 37% of men with PSA > 0.3 ng/mL versus 13% with a PSA < 0.3 ng/mL (*P* < .01) [29]. Tumor Gleason grade was not a statistically significant factor associated with local recurrence in this small cohort. In contrast, several surgical/clinical studies have shown that Gleason grade is predictive of the likelihood of BCR [13, 30–34] .

In the current study, with a larger number of patients (*n* = 195), a statistically significant relationship was observed between negative mpMRI findings and patients with lower Gleason grade tumors and PSA <  0.5 ng/mL. The majority of these studies (87.2%) were negative. This relationship was not observed for higher grade tumors. A statistically significant relationship was also observed between positive mpMRI results in patients with higher Gleason grade tumors and a PSA > 1.5 ng/mL, with the majority of these studies (88.9%) being positive.

To us, these findings suggest that, for patients with lower Gleason grade tumors and PSA < 0.5 ng/mL, the added value of mpMRI is low, since local recurrence will not be detected in a great majority of patients. At present, it is unclear if waiting for a PSA >  0.5 ng/mL to perform MR imaging would affect patient morbidity or mortality and may warrant further study.

For patients with higher Gleason grade tumors, PSA > 1.5 ng/mL, and clinical suspicion of local recurrence, mpMRI is also not very useful for detecting recurrence since a large majority (90%) will have recurrence on mpMRI. Instead, the added value of mpMRI in this clinical setting may be for treatment planning, including accurate target delineation for subsequent TRUS, MR-guided or MR/US fusion-guided biopsy for histologic confirmation and subsequent salvage therapy [3, 25, 35, 36] .

For patients with low post RP PSA (< 1.5 ng/mL) and higher Gleason grade tumors or lower Gleason grade tumors and PSA > 0.5 ng/mL, mpMRI results are less predictable. Thus, these populations of patients may benefit most from an mpMRI, both to confirm or exclude MR detectable local recurrence and for treatment planning, if indicated. Data from the current paper may also help guide when to perform post RP PET/MR studies [5], which can evaluate for local and distant disease, since mpMRI for local recurrence can complement radiotracer analysis [19].

There are limitations to this study, including that it is retrospective. Of note, all patients were included consecutively to remove bias. Interpretations of mpMRIs were used for statistical analyses. There are difficulties with a biopsy or follow-up reference standard including that most patients do not undergo biopsy of the prostatectomy bed or local lymph nodes, treatment is variable and may include no therapy or systemic therapy that may affect distant metastases in addition to or in the absence of local recurrence, and patients are often treated even in the absence of positive mpMRI findings limiting for example determination of false negatives. In this paper, we focused on the likelihood of mpMRI interpretation finding recurrence parsed by Gleason grade and PSA. Another limitation is that the study was performed at a single tertiary care institution focused on cancer. Study design concentrated on a clinical practice setting with interpretations from a group of fellowship trained radiologists focused on cancer with all clinical and laboratory data in the chart available reflecting a practice setting instead of one or a few expert readers. 3 T imaging was not utilized for this study; instead, mpMRI was performed at 1.5 T with placement of the endorectal coil near the area of interest to enable high resolution imaging. Future studies may employ 3 T MR with or without an endorectal coil, however, prior literature has not found an advantage of 3 T vs 1.5 T in this setting [37, 38] and both field strengths and an endorectal coil are used at various institutions [39]. Recurrence after other forms of therapy such as radiation or hormonal therapy was not evaluated, but could be studied in the future.

## Conclusion

In the setting of suspicion for local recurrence after RP, the findings from this study suggest that a large majority of mpMRI studies performed on patients with a PSA <  0.5 ng/mL and lower Gleason grade tumors are negative. In contrast, a majority performed on patients with a PSA > 1.5 ng/mL and higher Gleason grade tumors are positive. For patients with post RP PSA > 0.5 ng/mL and lower Gleason grade tumors or post RP PSA <  1.5 ng/mL and higher Gleason grade tumors, mpMRI results may be unpredictable, suggesting that these patients may benefit most from mpMRI for detecting recurrence after RP. For patients with PSA > 1.5 ng/mL and higher grade tumors, for whom most mpMRIs are positive, mpMRI may be more useful for directing biopsy and local therapy. These findings may help in selecting patients who may most benefit from an mpMRI in the setting of suspicion for local recurrence after RP to detect recurrence or to guide biopsy or treatment.

## Data Availability

The data is available to the journal for review and can be made available to others upon request.

## Abbreviations

mpMRI: Multiparametric MRI
RP: Radical prostatectomy
PSA: Prostate specific antigen
BCR: Biochemical recurrence
MR: Magnetic resonance
MRI: Magnetic resonance imaging
CT: Computed tomography
PET/CT: Positron emission tomography/ computed tomography
DCE: Dynamic contrast enhanced
DWI: Diffusion-weighted imaging
HIIPA: Health insurance portability and accountability act
T1W: T1- weighted
T2W: T2- weighted
FSE: Fast spin echo
ETL: Echo train length
rBW: Receiver bandwidth
FOV: Field of view
NEX: Number of excitations
RPART: Recursive partitioning and regression trees

## References

[1] American Cancer Society (2020). Cancer Facts & Figures.

[2] De Visschere PJL (2019). A systematic review on the role of imaging in early recurrent prostate Cancer. Eur Urol Oncol.

[3] American Urological Association Adjuvant and Salvage Radiotherapy after Prostatectomy:ASTRO/AUA Guideline (2013, amended 2018&2019). Available via https://www.auanet.org/guidelines/prostate-cancer-adjuvant-and-salvage-radiotherapy-guideline Accessed 19 Feb 2020*.*.

[4] Razek AA (2019). Whole-body diffusion-weighted imaging with background body signal suppression in the detection of osseous and extra-osseous metastases. Pol J Radiol.

[5] Bhargava P (2020). Imaging biochemical recurrence after prostatectomy: where are we headed?. AJR Am J Roentgenol.

[6] Gaur S, Turkbey B (2018). Prostate MR imaging for Posttreatment evaluation and recurrence. Radiol Clin N Am.

[7] Stephenson AJ (2006). Defining biochemical recurrence of prostate cancer after radical prostatectomy: a proposal for a standardized definition. J Clin Oncol.

[8] Amling CL (2001). Defining prostate specific antigen progression after radical prostatectomy: what is the most appropriate cut point?. J Urol.

[9] Khan MA, Partin AW (2004). Management of patients with an increasing prostate-specific antigen after radical prostatectomy. Curr Urol Rep.

[10] Panebianco V (2013). Prostate cancer recurrence after radical prostatectomy: the role of 3-T diffusion imaging in multi-parametric magnetic resonance imaging. Eur Radiol.

[11] Breiman L, Friedman J, Olshen RA, Stone CJ (1984). Classification and regression trees. Monterey.

[12] Han M (2003). Biochemical (prostate specific antigen) recurrence probability following radical prostatectomy for clinically localized prostate cancer. J Urol.

[13] Adamis S, Varkarakis IM (2014). Defining prostate cancer risk after radical prostatectomy. Eur J Surg Oncol.

[14] Heo JE (2020). Urethral realignment with maximal urethral length and bladder neck preservation in robot-assisted radical prostatectomy: urinary continence recovery. PLoS One.

[15] Freedland SJ (2006). Time to prostate specific antigen recurrence after radical prostatectomy and risk of prostate cancer specific mortality. J Urol.

[16] Pompe RS (2018). Long-term cancer control outcomes in patients with biochemical recurrence and the impact of time from radical prostatectomy to biochemical recurrence. Prostate.

[17] Antonarakis ES (2012). The natural history of metastatic progression in men with prostate-specific antigen recurrence after radical prostatectomy: long-term follow-up. BJU Int.

[18] Evans JD (2018). Prostate cancer-specific PET radiotracers: a review on the clinical utility in recurrent disease. Pract Radiat Oncol.

[19] Guberina N, et al. Whole-body integrated [(68) Ga]PSMA-11-PET/MR imaging in patients with recurrent prostate Cancer: comparison with whole-body PET/CT as the standard of reference. Mol Imaging Biol. 2019. 10.1007/s11307-019-01424-4 31482413

[20] Sharma V (2018). Multiparametric magnetic resonance imaging is an independent predictor of salvage radiotherapy outcomes after radical prostatectomy. Eur Urol.

[21] Alfarone A (2012). Comparative analysis of multiparametric magnetic resonance and PET-CT in the management of local recurrence after radical prostatectomy for prostate cancer. Crit Rev Oncol Hematol.

[22] Sella T (2004). Suspected local recurrence after radical prostatectomy: endorectal coil MR imaging. Radiology.

[23] Sciarra A (2008). Role of dynamic contrast-enhanced magnetic resonance (MR) imaging and proton MR spectroscopic imaging in the detection of local recurrence after radical prostatectomy for prostate cancer. Eur Urol.

[24] Panebianco V (2012). Prostate cancer: 1HMRS-DCEMR at 3T versus [(18) F] choline PET/CT in the detection of local prostate cancer recurrence in men with biochemical progression after radical retropubic prostatectomy (RRP). Eur J Radiol.

[25] Cirillo S (2009). Endorectal magnetic resonance imaging at 1.5 tesla to assess local recurrence following radical prostatectomy using T2-weighted and contrast-enhanced imaging. Eur Radiol.

[26] Casciani E (2008). Endorectal and dynamic contrast-enhanced MRI for detection of local recurrence after radical prostatectomy. AJR Am J Roentgenol.

[27] Counago F (2015). Role of 3T multiparametric magnetic resonance imaging without endorectal coil in the detection of local recurrent prostate cancer after radical prostatectomy: the radiation oncology point of view. Scand J Urol.

[28] Kitajima K (2015). Detection of local recurrence of prostate Cancer after radical prostatectomy using Endorectal coil MRI at 3 T: addition of DWI and dynamic contrast enhancement to T2-weighted MRI. AJR Am J Roentgenol.

[29] Liauw SL (2013). Evaluation of the prostate bed for local recurrence after radical prostatectomy using endorectal magnetic resonance imaging. Int J Radiat Oncol Biol Phys.

[30] Han M (2001). Long-term biochemical disease-free and cancer-specific survival following anatomic radical retropubic prostatectomy. The 15-year Johns Hopkins experience. Urol Clin North Am.

[31] Hull GW (2002). Cancer control with radical prostatectomy alone in 1,000 consecutive patients. J Urol.

[32] Kupelian P (1996). Correlation of clinical and pathologic factors with rising prostate-specific antigen profiles after radical prostatectomy alone for clinically localized prostate cancer. Urology.

[33] Mian BM (2002). Outcome of patients with Gleason score 8 or higher prostate cancer following radical prostatectomy alone. J Urol.

[34] Wiegel T (2009). Phase III postoperative adjuvant radiotherapy after radical prostatectomy compared with radical prostatectomy alone in pT3 prostate cancer with postoperative undetectable prostate-specific antigen: ARO 96-02/AUO AP 09/95. J Clin Oncol.

[35] Menard C (2015). MR-guided prostate biopsy for planning of focal salvage after radiation therapy. Radiology.

[36] Muller BG (2015). Multiparametric magnetic resonance imaging-transrectal ultrasound fusion-assisted biopsy for the diagnosis of local recurrence after radical prostatectomy. Urol Oncol.

[37] Buergy D (2018). Detection of local recurrence with 3-tesla MRI after radical prostatectomy: a useful method for radiation treatment planning?. In Vivo.

[38] Potretzke TA, Froemming AT, Gupta RT (2020). Post-treatment prostate MRI. Abdom Radiol (NY).

[39] Jambor I (2020). Prediction of biochemical recurrence in prostate cancer patients who underwent prostatectomy using routine clinical prostate multiparametric MRI and decipher genomic score. J Magn Reson Imaging.

